# Osteoporosis: Pathophysiology and therapeutic options

**DOI:** 10.17179/excli2020-2591

**Published:** 2020-07-20

**Authors:** Ursula Föger-Samwald, Peter Dovjak, Ursula Azizi-Semrad, Katharina Kerschan-Schindl, Peter Pietschmann

**Affiliations:** 1 Institute of Pathophysiology and Allergy Research, Center for Pathophysiology, Infectiology and Immunology, Medical University of Vienna, Vienna, Austria; 2 Department of Acute Geriatrics, Salzkammergut Klinikum Gmunden, Gmunden, Austria; 3 Department of Emergency Medicine, Medical University of Vienna, Vienna, Austria; 4 Department of Physical Medicine, Rehabilitation and Occupational Medicine, Medical University of Vienna, Vienna, Austria

**Keywords:** osteoporosis, pathophysiology, osteoimmunology, gut microbiome, senescence, osteoporosis treatment

## Abstract

Osteoporosis is a metabolic bone disease that, on a cellular level, results from osteoclastic bone resorption not compensated by osteoblastic bone formation. This causes bones to become weak and fragile, thus increasing the risk of fractures. Traditional pathophysiological concepts of osteoporosis focused on endocrine mechanisms such as estrogen or vitamin D deficiency as well as secondary hyperparathyroidism. However, research over the last decades provided exiting new insights into mechanisms contributing to the onset of osteoporosis, which go far beyond this. Selected mechanisms such as interactions between bone and the immune system, the gut microbiome, and cellular senescence are reviewed in this article. Furthermore, an overview on currently available osteoporosis medications including antiresorptive and bone forming drugs is provided and an outlook on potential future treatment options is given.

## Introduction

Osteoporosis, the most frequent form of metabolic bone diseases, is defined as a ”skeletal disorder characterized by compromised bone strength predisposing a person to an increased risk of fracture”. Furthermore, bone strength is defined to “primarily reflect the integration of bone density and bone quality” (NIH Consensus Development Panel on Osteoporosis Prevention, Diagnosis, and Therapy, 2001[85]). Although osteoporosis can occur at any age and in both genders, it typically is an age related disease that more frequently affects women than men. In contrast to other musculoskeletal diseases such as osteoarthritis or sarcopenia, for osteoporosis effective treatment options that interfere with the underlying disease processes are available; nevertheless, in clinical reality only a relatively small fraction of patients is treated adequately. With the aging of our societies, it is very likely that the number of patients suffering from osteoporosis will increase dramatically; thus, intensive further research to identify novel therapeutic targets urgently is needed. This article is intended to contribute to this goal in reviewing disease mechanisms, the current and potential future treatment options for osteoporosis. Based upon our personal expertise, a special focus will be given to osteoimmunology.

## Bone Biology

Research over the years has evidenced the central role of bone as an organ that is in constant exchange with and regulates several other tissues. Accordingly, it is now well accepted that bone, in addition to its classical roles in locomotion, protection of internal organs, and regulation of mineral homeostasis, contributes to the regulation of glucose metabolism and energy expenditure and influences male fertility and cognitive functions through the secretion of osteocalcin by osteoblasts (Ponzetti and Rucci, 2019[100]; Wei and Karsenty, 2015[128]). In order to perform its diverse functions, bone undergoes continuous cycles of modeling and remodeling. During **modeling** either bone formation or bone resorption occur independently at distinct sites. Thereby, changes in dimensions and shape of bone during growth and adaption of bone to altering mechanical demands are facilitated. **Remodeling**, in contrast, is a highly coordinated process of concomitant resorption and formation at a distinct site and is responsible for the maintenance of skeletal integrity by renewing old and damaged bone. Additionally, remodeling processes maintain calcium and phosphate homeostasis by targeted release and incorporation from and into the bone matrix. The crucial role of remodeling in overall bone homeostasis is highlighted by the fact that impaired remodeling favoring bone resorption over bone formation is a fundamental pathophysiological mechanism leading to bone pathologies such as osteoporosis.

**Figure 1:**
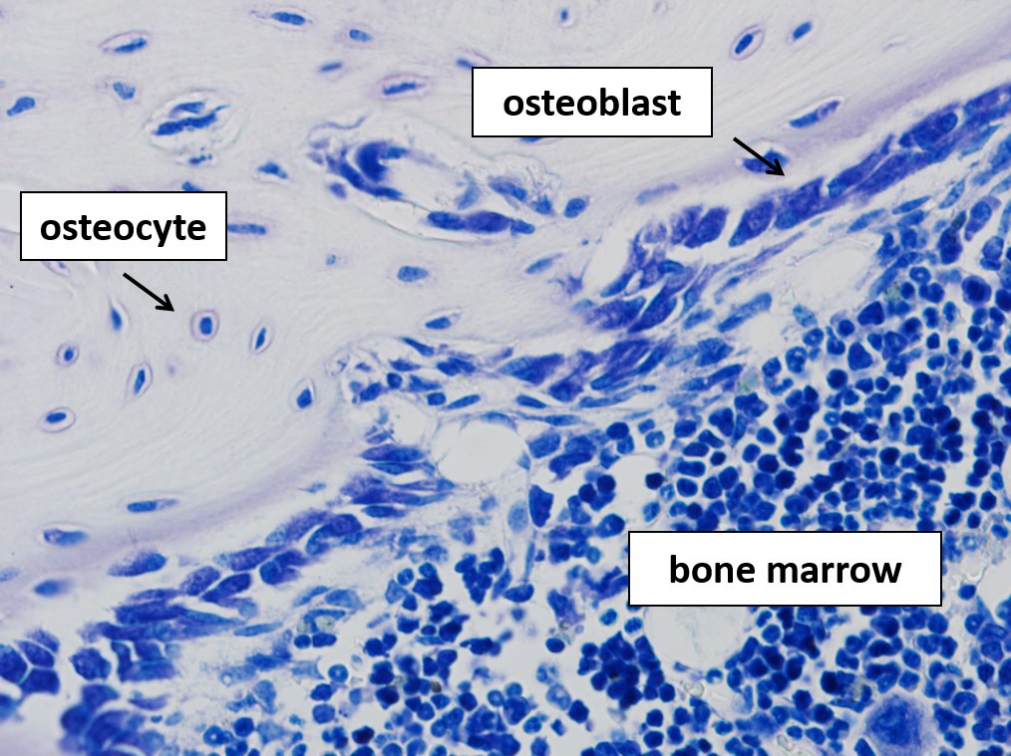
Histological section of a mouse femur; stain: toluidin blue; original magnification: 400X

Key cellular components in bone modeling and remodeling are three types of **bone cells**: bone resorbing osteoclasts, bone forming osteoblasts, and osteocytes, former osteoblasts that have become trapped in the bone matrix (Figure 1(Fig. 1)). In particular remodeling depends on a fine tuned crosstalk between these protagonists to ensure that the amount of bone resorbed by osteoclasts equals the amount of bone formed by osteoblasts and thereby, to ensure the maintenance of bone mass. A major step forward in understanding this “coupling” process was the discovery of receptor activator of nuclear factor-kB (RANK), its ligand **RANKL** and its decoy receptor **osteoprotegerin** (OPG). RANKL was initially found to be expressed by osteoblasts and its progenitors and, together with macrophage colony-stimulating factor (M-CSF), is regarded as a master regulator of osteoclast survival, activation, and differentiation from hematopoetic linage cells (Boyce and Xing, 2008[19]). In addition to osteoblasts and bone marrow stromal cells, RANKL is expressed also by osteocytes and by various extraskeletal cells and tissues including cells of lymphoid tissues (Leibbrandt and Penninger, 2008[72]; Nakashima et al., 2011[83]). The role of RANKL as a key regulator of bone homeostasis is strengthened by the fact that many other cytokines known to influence bone resorption do so by indirectly manipulating RANKL signaling. Amongst these are **proinflammatory cytokines** such as interleukin 1 (IL-1), and tumour necrosis factor (TNF), which already in the 1980s have been demonstrated to stimulate bone resorption (Bertolini et al., 1986[11]; Gowen et al., 1983[56]). Thereby, a clear connection between the skeletal and the immune system has been established which, in the last two decades, was further complemented by the pioneering work on the role of T-helper 17 (Th17) cells in osteoimmunology (Sato et al., 2006[110]; Tsukasaki and Takayanagi, 2019[124]). This subset of T-helper cells is characterized by the expression of the osteoclastogenesis inducing cytokine interleukin 17 (IL-17) and is now well known to contribute to bone loss seen in inflammatory conditions such as rheumatoid arthritis (Tsukasaki and Takayanagi, 2019[124]). Master regulators of **osteoblast** differentiation are bone morphogenetic proteins (BMP) and wingless-related integration site (Wnt) signaling pathway proteins, which, by binding to the transmembrane proteins frizzeld receptor (Fzd) and LDL receptor-related protein (LRP) 5 or 6, trigger the canonical Wnt signaling pathway (Lin and Hankenson, 2011[75]). The molecule Sclerostin (SOST) is an inhibitor of Wnt signaling. Absence of SOST due to loss of function mutations in van Buchem disease and sclerostosis is linked to a high bone mass phenotype, thereby evidencing a crucial role of this pathway in osteoblast physiology (de Vernejoul and Kornak, 2010[41]). 

**Osteocytes** are the most abundant cell type in bone and were for a long time regarded as non-participating bystanders of bone metabolism. However, new insights into osteocyte physiology during the last two decades support a pivotal role of this cell type in bone and mineral homeostasis. Besides being important regulators of calcium and phosphate homeostasis, their central role as initiators and drivers of bone remodeling by communicating with and orchestrating osteoblast and osteoclast formation and activity is now well established (Schaffler et al., 2014[111]). 

This crosstalk is facilitated by a complex network of dendritic processes, which spans the whole bone matrix. It connects osteocytes with the bone surface and the vasculature. Thereby, osteocytes directly or by the release of effector proteins influence osteoclast and osteoblast activity on the surface of a basic multicellular unit, a temporary anatomic structure where bone is remodeled. Important effector proteins released by osteocytes and modulating osteoblast and osteoclast formation are SOST, an inhibitor of the Wnt signaling pathway, and RANKL, respectively (Dallas et al., 2013[36]). The dogma of osteocytes being passive bystanders of metabolism was further challenged by the finding that osteocytes are capable of bone destruction in a process termed osteocytic osteolysis, and of depositing new bone material in the vicinity of osteocytes. Thereby, they are involved in remodeling their immediate surrounding bone matrix in various physiological and pathophysiological conditions including immobilization, hyperparathyroidism, and lactation (Tsourdi et al., 2018[123]).

## Pathophysiology of Osteoporosis

**Figure 2:**
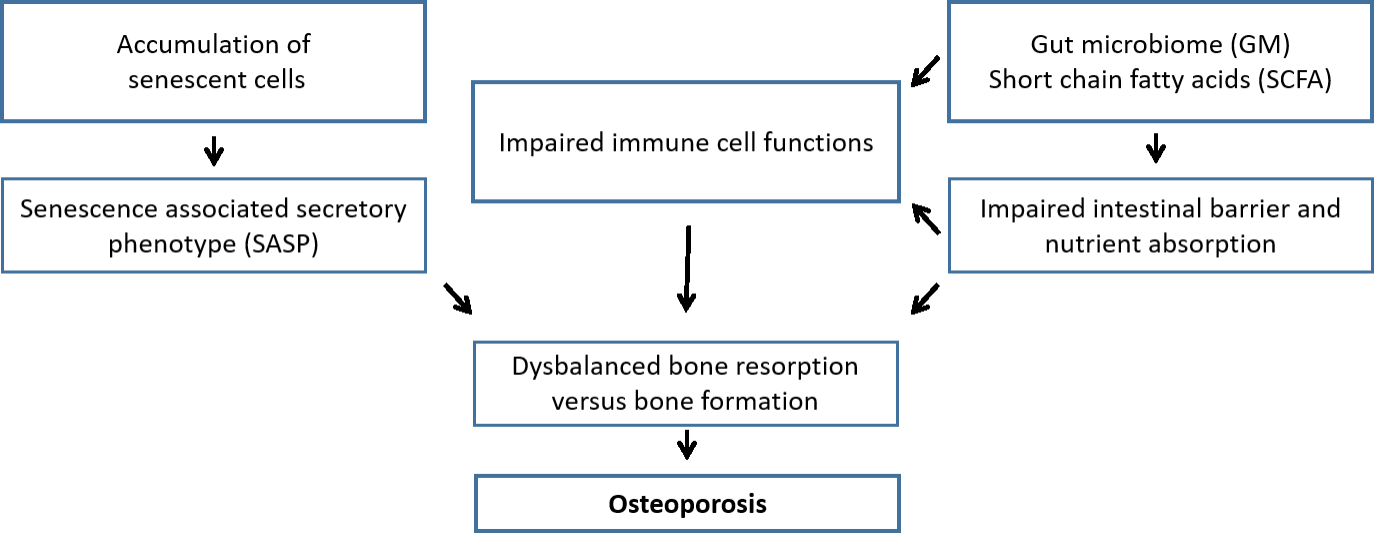
Overview on pathophysiological mechanisms discussed in this review

Osteoporosis is a classic example of a multifactorial disease with a complex interplay of genetic, intrinsic, exogenous, and life style factors contributing to an individual's risk of the disease. Traditional pathophysiologic models frequently emphasized endocrine mechanisms, e.g. estrogen deficiency and secondary hyperparathyroidism in elderly due to estrogen deficiency, reduced dietary intake, and widely prevalent vitamin D deficiency, as the key determinants of postmenopausal osteoporosis (Clarke and Khosla, 2010[28]). However, it has become clear in the last years that pathophysiological mechanisms contributing to the onset of osteoporosis go far beyond this. Selected mechanisms will be discussed in the following paragraphs (Figure 2(Fig. 2)).

### Osteoimmunology

An emerging role in bone pathophysiology has been attributed to the immune system, giving rise to a new field of research termed osteoimmunology. The concept of osteoimmunology refers to the mutual interactions between the immune system and bone. In the English literature the term “osteoimmunology” was coined in 2000 by Arron and Choi (2000[6]); nevertheless, one of the authors of this review article (PP) already had used the German term “Osteoimmunologie” in 1997.

At the cellular level, the **osteoclast**, the cell responsible for bone resorption, can be regarded as the prototype of an osteoimmune cell: osteoclasts share common precursor cells with monocytes, macrophages, and (myeloid) dendritic cells. Accordingly, first insights into the osteoimmunological crosstalk were gained by studies on interactions of immune cells and osteoclasts leading to bone destruction in inflammatory diseases such as periodontitis or rheumatoid arthritis (Horton et al., 1972[61]). By now, it has become clear that cells of the immune and bone system have many molecules, such as transcription factors, signaling factors, cytokines, or chemokines, in common (Tsukasaki and Takayanagi, 2019[124]). One of the first type of immune cells that have been shown to mediate effects of the immune system on bone are T cells, in particular CD4^+^ cells. In **rheumatoid arthritis**, a typical osteoimmune disorder characterized by bone erosions in multiple joints in conjunction with inflammation of the synovium, stimulation of bone resorption by osteoclast is exclusively mediated by the **Th17 subset of CD4^+^ cells** (Kotake et al., 1999[71]; Sato et al., 2006[110]). These cells accumulate in the synovial fluid of patients with RA and promote osteoclastogenesis in the first place by the secretion of IL-17 that stimulates RANKL expression by syno-vial fibroblasts (Hirota et al., 2007[59]; Kontake et al., 1999[71]; Nistala et al., 2008[86]; Sato et al., 2006[110]). Additionally, IL-17 augments local inflammation. Hence, the production of inflammatory cytokines such as TNF and Il-6 is increased, which in turn amplifies RANKL expression and thereby indirectly fuels osteoclastogenesis (Sato et al., 2006[110]). In recent years, the most potent pro-osteoclastogenic CD4^+^ subset was further narrowed down to a particular type of Th17 cells, which derives from FOXP3^+^ T cells. They have been shown to loose expression of the transcription factor FOXP3 under arthritic conditions, thereby promoting the development of Th17 instead of regulatory T-cells (T_reg_) (Komatsu et al., 2014[70]).

To sum up, tremendous progress has been made in elucidating protagonists and pathways contributing to bone destruction in conjunction with inflammatory diseases, in particular with rheumatoid arthritis (Tsukasaki and Takayanagi, 2019[124]). The contribution of the immune system to bone loss seen in osteoporosis, in contrast, is less well understood. Cessation of ovarian function and, associated therewith, loss of estrogen has been known since nearly eight decades to be a key event in promoting accelerated bone loss in early menopause (Albright et al., 1941[3]), and, as proposed by the unitary model for the pathophysiology of primary or involutional osteoporosis, also in slow bone loss in late post menopause, referred to as age-related bone loss, and in elderly men (Riggs et al., 1998[104]). Effects of estrogen loss are to some extend mediated by a direct modulation of osteoblast, osteoclast, and osteocyte physiology via estrogen receptors on these cells. Specifically, estrogen loss increases the number of osteoclasts and at the same time decreases the number of osteoblasts leading to an unbalanced activity of the basic multicellular unit in favor of bone resorption (Clarke and Khosla, 2010[28]). However, it is now well accepted, that effects of estrogen deficiency, in particular on bone resorption, are mainly indirect via the release of bone-active cytokines. Among these osteoclastogenic cytokines are inflammatory cytokines, suggesting a role of interactions of the immune system and bone tissue also in the pathophysiology of osteoporosis. A role of proinflammatory cytokines in osteoporosis is strengthened by the findings of elevated levels of TNF, IL-1, IL-6, or IL-17 in the first ten years after menopause (Pacifici et al., 1990[90]) and in osteoporotic postmenopausal women compared to non-osteoporotic postmenopausal women (Zhao et al., 2016[135]). Moreover, the phenomena of “inflammaging” characterized by a low-grade inflammatory status and increased levels of proinflammatory markers in elderly people (Franceschi et al., 2000[48]) has in several studies been linked to bone loss and fracture risk (Cauley et al., 2007[22]; Ding et al., 2008[42]; Ganesan et al., 2005[51]; Nakamura et al., 2011[82]; Pasco et al., 2006[95]). Likewise, chronic inflammatory diseases, e.g. rheumatoid arthritis or Crohn's disease, promote an osteoporotic phenotype (Blaschke et al., 2018[15]; Sapir-Koren and Livshits, 2017[109]). 

As in rheumatoid arthritis, also in osteoporosis T-cells are thought to be a major source of proinflammatory cytokines (Pietschmann et al., 2016[98]; Rauner et al., 2007[102]). Our group has shown that the proportion of CD8^+^ cells that express TNF is expanded in postmenopausal women with osteoporotic fractures when compared to age-matched controls (Pietschmann et al., 2001[97]). D'Amelio et al. described a higher production of TNF in T-cells and monocytes (D'Amelio et al., 2008[37]) and Zhao et al. an upregulated expression of IL-17 in CD4^+^ cells of osteoporotic postmenopausal women (Zhao et al., 2016[135]). However, an important link between estrogen loss, T-cell-dependent inflammation, and osteoporosis has been unraveled only recently. Cline-Smith et al. for the first time described a molecular mechanism by which estrogen loss promotes a low-grade inflammation by T-cells during the acute phase of bone loss in ovariectomized mice (Cline-Smith et al., 2020[29]). They provide data suggesting a central role of **bone marrow dendritic cells** (BMDCs) in the induction of proinflammatory cytokines producing T-cells. Ovariectomy (OVX) in their study increases the number of BMDCs and subsequently the amount of IL-7 and IL-15 produced by these cells. IL-7 and IL-15, in turn, induces antigen-independent production of IL-17A and TNF in a subset of memory T cells (T_MEM_). Further, a crucial role of the suggested pathway in OVX associated bone loss was confirmed by showing that T-cell-specific ablation of IL15RA prevented IL-17A and TNF expression and an increase in bone resorption or bone loss after OVX (Cline-Smith et al., 2020[29]). Interestingly, the authors speculate that a crucial role of the activation of T_MEM_ in postmenopausal bone loss may provide an explanation for varying susceptibilities to develop osteoporosis within a population. Varying levels of T_MEM_ in individuals reflect different lifetime exposures to commensal and pathogenic microbes and might be associated with higher or lower levels of TNFα and IL-17A and in consequence of bone loss induced after menopause (Cline-Smith et al., 2020[29]).

Another subclass of T-cells increasingly evidenced to act at the interface of the immune and skeletal system are **regulatory T** (T_reg_) cells. They are characterized by the expression of the transcription factor FOXP3 and their main function is to suppress multiple types of immune cells and prevent excessive immune reactions, inflammation, and tissue damage. Their role in bone biology is clearly anti-osteoclastogenic (Bozec and Zaiss, 2017[20]). Accordingly, the transfer of T_reg_ cells into T-cell deficient mice was associated with an increased bone mass and a decreased number of osteoclasts (Zaiss et al., 2010[134]). Moreover, *Foxp3* transgenic mice were protected from OVX-induced bone loss, supporting a role of T_reg_ cells in bone loss in conjunction with estrogen deprivation (Zaiss et al., 2010[134]) Consistent with this, estrogen has been shown to stimulate proliferation and differentiation of T_reg_ cells (Tai et al., 2008[117]).

A role of **B-cells** in the pathophysiology of osteoporosis is supported by the finding that they produce RANKL and OPG, and, hence, act as regulators of the RANK/ RANKL/OPG axis (Walsh and Choi, 2014[127]). Indeed, production of RANKL by B-cells is increased in postmenopausal women (Eghbali-Fatourechi et al., 2003[45]) and B-cell ablation of RANKL in mice partially protects from trabecular bone loss after ovariectomy (Onal et al., 2012[88]). A role of B-cells in bone metabolism and osteoporosis is further strengthened by the results of a global gene expression study by Pineda et al.. Comparing gene expression in OVX mice and control mice they identified several pathways attributed to B-cell biology among the top canonical pathways affected (Pineda et al., 2014[99]). A more recent study compared global gene expression in B-cells obtained from the bone marrow of OVX and control mice (Panach et al., 2017[92]). In a second stage, they studied the association of polymorphisms in selected differentially expressed genes in postmenopausal women and identified a significant association of single nucleotide polymorphisms (SNPs) in CD80 with bone mineral density (BMD) and the risk of osteoporosis. A possible link between this molecule and BMD might be indirect via its costimulatory function for the activation of T-cells or direct via the described inhibitory effect on osteoclast generation (Bozec et al., 2014[21]). To sum up, substantial evidence for a contribution of B-cells to the development of osteoporosis exists. However, the exact mechanism linking estrogen deficiency to B-cells and bone loss seen in postmenopausal women remains incompletely understood.

### Gut microbiome and osteoporosis

A novel and rapidly expanding field deals with the influence of the gut microbiome (GM) on a person's health and provides exciting new insights into the crosstalk between the homeostasis of bone metabolism and the intestinal flora (Behera et al., 2020[10]; Ding et al., 2020[43]; Pacifici, 2018[90]). It is now well accepted that the GM, the entirety of microorganism living in the human digestive tract, influences development and homeostasis of gastrointestinal (GI) tract tissues and also of tissues at extra-GI sites (e.g nutrient production and absorption, host growth, immune homeostasis). Moreover, complex diseases such as type 1 and 2 diabetes, transient ischemic attack, or rheumatoid arthritis have been linked to changes in the composition of the GM (Behera et al., 2020[10]). Sjogren et al. have shown that germ-free mice exhibit increased bone mass and thereby first evidenced a relation between bone homeostasis and the GM (Sjogren et al., 2012[113]). Additional support for this crosstalk comes from experimental data showing that modulation of the GM by the use of **probiotics** or **antibiotics** affects bone health (Guss et al., 2019[57]; Li et al., 2016[73]; Ohlsson et al., 2014[87]; Parvaneh et al., 2015[94]; Rozenberg et al., 2016[106]). An important evidence for a role of the GM in estrogen driven bone loss comes from a study showing that germ-free mice are protected from trabecular bone loss induced by sex steroid deprivation (Li et al., 2016[73]).

Various mechanisms have been proposed to modulate this close “**microbiota-skeletal**” axis, one of them being the effects of the GM on host metabolism. The GM has been shown to influence the absorption of nutrients required for skeletal development such as calcium, and thereby affect bone mineral density (Rodrigues et al., 2012[105]). Absorption of nutrients might be influenced by intestinal pH values, which depend on the composition of the GM. Additionally, microbial fermentation of dietary fibers to **short chain fatty acids** (SCFAs) seems to play an important role in this process. In adults, consumption of different prebiotic diets that can be fermented to SCFAs was associated with an increased resorption of calcium (Whisner et al., 2014[129], 2016[130]). Beyond this influence on intestinal nutrient absorption, SCFAs have emerged as potent regulators of osteoclast differentiation and activity and of bone metabolism (Zaiss et al., 2019[133]). For instance, in mice fed with SCFAs or a high-fiber-diet an increase in bone mass was observed. Moreover, postmenopausal as well as inflammation-induced bone loss was prevented and the protective effect was associated with impaired osteoclast differentiation and bone resorption (Lucas et al., 2018[77]). SCFAs are therefore an example of gut-derived microbial metabolites that diffuse into the systemic circulation. By doing so, these substances can regulate anatomically distant organs such as the skeletal system (Zaiss et al., 2019[133]). 

A well-established function of the GM is to modulate immune functions. Hence, effects of the GM on intestinal and systemic immune responses, which in turn modulate bone homeostasis, provide another important link between the GM and the skeletal system. Bone active cytokines released by immune cells in the gut or immune cells activated in the gut and then circulating to the bone are discussed as mechanisms most likely mediating this GM-immune-bone axis (Pacifici, 2018[90]). Among cells of the immune system, Th17 cells and T_reg _cells are thought to play a prominent role in this crosstalk. Specifically, the balance of Th17/T_reg_ cells has been shown to be modulated by gut macrobiotics (Dar et al., 2018[39][40]), and also here a key role in promoting the differentiation and proliferation of T_reg_ cells is attributed to SCFAs (Arpaia et al., 2013[5]; Furusawa et al., 2013[49]; Smith et al., 2013[114]; Zaiss et al., 2019[133]).

Another unexpected link between SCFAs, the immune system, and bone metabolism was reported only recently. Li et al. demonstrated that the bone formation stimulating effect of intermittent **parathyroid hormone** (PTH) treatment depends on SCFAs, in particular butyrate, produced by the microbiome (Li et al., 2020[74]). Further, they provided evidence for butyrate, in concert with PTH, to induce CD4^+^ T cells to differentiate into T_reg_ cells, which in turn stimulate CD8^+^ T cells to produce Wnt10b (Li et al., 2020[74]). Wnt10b is a key activator of Wnt signaling in stromal cells and osteoblasts and is known to promote bone formation by increasing osteoblast proliferation, differentiation, and survival (Monroe et al., 2012[80]). Also PTH induced bone loss has been known to depend on T cell activation (Gao et al., 2008[53]; Tawfeek et al., 2010[118]), but it was not clear if these T-cells originate in the bone marrow or in the intestine. Only recently, it was shown by Yu et al. that bone loss induced by PTH depends on activation of intestinal TNF^+^ and Th17 T cells in response to the gut microbiota and recruitment of these cells to the bone marrow (Yu et al., 2020[132]). Taken together, increasing evidence attributes the microbiome and metabolites produced by the microbiome, in particular SCFAs, a key regulatory function in bone homeostasis. Probiotics and interventions targeting the GM and its metabolites might therefore be a promising future strategy for the prevention and treatment of osteoporosis.

### Cellular senescence and osteoporosis

Cellular senescence describes a cell fate induced by various types of stress and is associated with irreversible cell cycle arrest and resistance to apoptosis (Hayflick, 1965). In addition, cells entering senescence are characterized by excessive production of proinflammatory cytokines, chemokines, and extracellular matrix-degrading proteins, a state referred to as **senescence-associated secretory phenotype** (SASP) (Tchkonia et al., 2013[120]). The number of senescent cells increases during the process of aging (Tchkonia et al., 2010[119]), which has been evidenced to play a major role in age-related tissue dysfunction and the development of several age-related diseases such as diabetes mellitus, hypertension, atherosclerosis, or osteoporosis (Khosla et al., 2020[68]).

An important contribution to understanding the role of senescence in the development of osteoporosis was made by Farr et al. only a few years ago. They have shown that B cells and T cells, myeloid cells, osteoprogenitors, osteoblasts, and osteocytes are among the types of cells within the bone microenvironment becoming senescent with aging, and that an increased production of key SASP factors with aging can be seen particularly in senescent myeloid cells and osteocytes (Farr et al., 2016[46]). Further, they provided evidence for an accumulation of senescent cells in bone biopsy samples from older postmenopausal women compared to younger premenopausal women (Farr et al., 2016[46]). In a more recent study, Farr et al. provided a causative link between cellular senescence and age-related bone loss. They demonstrated that elimination of senescent cells or inhibition of their SASP prevented age-related bone loss in mice by decreasing trabecular and cortical bone resorption and increasing or maintaining bone formation on endocortical and trabecular surfaces, respectively (Farr et al., 2017[47]). Further, consistent with the finding of an increased SASP particularly in osteocytes (Farr et al., 2016[46]; Piemontese et al., 2017[96]), the observed bone sparing effect was mediated partly by the elimination of senescent osteocytes (Farr et al., 2017[47]). To sum up, considerable progress has been made in understanding the role and mechanisms of senescence in age-related bone loss. Based on these new findings, targeting cellular senescence by senolytics and senostatics has emerged as a potential promising therapeutic strategy for the treatment of age-related osteoporosis. 

## Treatment Options

### Interlude

The goals of osteoporosis therapy are to reduce fracture risk and bone loss, prevent disability, and control pain (Spencer, 1982[115]). Prevention strategies include fall reduction, correcting impaired vision or hearing, reducing fall risk inducing drugs (FRIDs), establishing muscle training, balance training, quit cigarette smoking and alcohol intake, and adequate intake of vitamin D, protein and calcium (Akkawi and Zmerly, 2018[1]). An impressive number of evidence has been published in the last years with enormous impact on clinical application. Therefore, it seems reasonable to continue this narrative review on current treatment options (for an overview see Table 1(Tab. 1); References in Table 1: Bischoff-Ferrari et al., 2009[12]; Black and Rosen, 2016[14]; Chesnut et al., 2004[27]; Compston et al., 2017[30]; Cosman et al., 2016[33]; Cummings et al., 2009[35]; D'Amelio and Isaia, 2013[38]; Kanis et al., 2013[65]; Neer et al., 2001[84]; Reid et al., 2009[103]).

The treatment for osteoporosis in the first step consists of basic interventions such as prescribing exercise, weight-bearing physical activity and exercises that improve balance and posture, a diet rich in vitamin D and calcium, quitting smoking, and limiting alcohol use, measures summarized under point “Nonpharmacological treatment” In the second step, specific medication prescriptions are necessary (Figure 1(Fig. 1)). Medication groups are divided in anti-resorptive (anti-catabolic) drugs, anabolic drugs, and combinations of remedies (Ukon et al., 2019[125]). Osteoclasts remain the main targets of medical intervention, even though osteoblasts emerge as new cells of interest in that topic. The Wnt signaling pathway has become a fascinating spot for new medical interventions; Dickkopf-1 and sclerostin, both inhibitors of this pathway, are promising therapeutic targets (Chen et al., 2019[25]). 

Nevertheless, only a minor part of eligible patients is treated with any of these therapeutic options. In a population-based study published by Lorentzon et al. only a proportion of 21,8 % received an adequate treatment (Lorentzon et al., 2019[76]). Non-adherence is particularly to blame for the undertreatment and therefore of interest. Main reflections on adherence are mentioned under the heading “Adherence”.

### Basic medication

A protective effect against hip and non-vertebral fractures can be achieved with vitamin D doses ≥ 800 international units (IU) daily in combination with a calcium intake between 700 and 1200 mg/day (preferable by dietary intake). This dose of vitamin D, effective in reducing the risk of falls, is recommended in women and men ≥ 50 years, and can be increased in patients with higher risk of fractures. However, the intermittent prescription of large doses of vitamin D (≥ 100.000 IU) is associated with an increased risk of fractures and falls (Sanders et al., 2010[108]). The combination of calcium and vitamin D is necessary in patients treated with bone specific therapies (to achieve the full effects shown in the intervention trials) and to prevent secondary hyperparathyroidism, hypomagnesemia, and disturbances of bone metabolism (Bolland et al., 2015[16]; Compston et al., 2017[30])*.* Vitamin D is fat-soluble; for optimal resorption it is important to provide it with the meal.

### Specific medication

**Figure 3:**
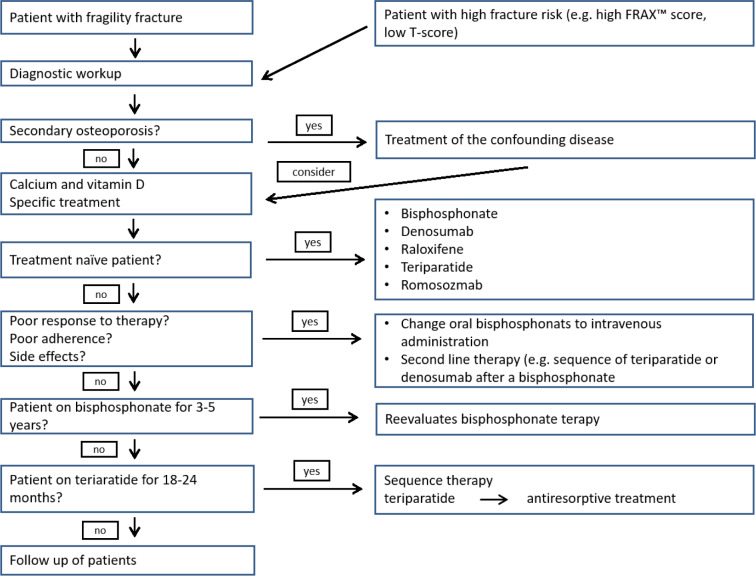
Algorithm for the diagnosis and pharmacologic treatment of osteoporosis (adapted from Kanis et al., 2019, Cosman, 2020 and Anastasilakis et al., 2020)

Based on clinical algorithms published in guidelines (Figure 3(Fig. 3); References in Figure 3: Kanis et al., 2019[64]; Cosman, 2020[32]; Anastasilakis et al., 2020[4]), specific medication must be offered to patients with a high risk of fractures or patients with a fragility fracture (Kanis et al., 2019[64]; Qaseem et al., 2017[101]). No study has been powered to show differences in risk reduction of fractures between the different treatment modalities. Nevertheless, osteoanabolic drugs reduce the risk of vertebral and nonvertebral fractures in a faster mode than antiresorptive drugs and should be considered as first line therapy in special clinical circumstances such as in patients with prior fragility fractures, multiple fractures during the clinical course (Cosman, 2020[32]), and a very low bone mineral density (t-score below - 3) (Cosman, 2020[32]). In general, the choice of treatment is made on clinical judgement considering potential side effects, effects on different skeletal parts, and costs.

#### Bisphosphonates

Bisphosphonates inhibit bone resorption by attaching to hydroxyapatite binding sites on bone surfaces during active resorption. This hampers osteoclasts forming the border, the adherence to the bone surface, and the production of protons necessary for their action. They also reduce osteoclast progenitor development and recruitment and promote osteoclast apoptosis (Hughes et al., 1995[62]). Furthermore, bisphosphonates have an effect on osteoblasts. However, this effect does not contribute significantly to the efficacy of bisphosphonates. 

**Alendronate** must be taken after an overnight fast and 30 minutes before breakfast (and the intake of other drugs) or drinks (other than water) once weekly 70 mg by mouth or 10 mg daily. The medical indications include the prevention of postmenopausal osteoporosis and glucocorticoid-induced osteoporosis (in a reduced dose of 5 mg daily), and the treatment of postmenopausal osteoporosis, osteoporosis in men, and glucocorticoid induced osteoporosis. Aledndronate has been shown to prevent vertebral and hip fractures (Black and Rosen, 2016[14]). After the ingestion of the drug, the patient should be in an upright position for 30 minutes to minimize the risk of upper gastrointestinal side effects. 

**Risedronate** is approved for the treatment of postmenopausal osteoporosis and for men with osteoporosis and a high risk for fractures, as well as for the prevention of fractures in men and postmenopausal women taking glucocorticoids. The 5 mg preparation daily and the 35 mg preparation once weekly per mouth have been shown to reduce vertebral and hip fractures and should be taken in the same manner as alendronate (Kanis et al., 2013[65]).

Oral preparations are associated with a high number of non-adherence and reduced treatment effects due to side effects, low absorption of the oral preparation, and the manner of administration. Furthermore, comorbidities, polypharmacy, and functional decline in elderly patients account for this drawback (Gamboa et al., 2018[50]).

**Zoledronic acid** 5 mg intravenously over a period of 15 minutes once a year has been approved for the treatment of osteoporosis in postmenopausal women and men with increased risk of fractures, and in women and men taking long- term glucocorticoids. Flu-like symptoms after the first infusion were reported in 4.7 % of patients; these side effects can be reduced by giving acetaminophen before the infusion (Reid et al., 2009[103]).

**Ibandronate** is prescribed once monthly by mouth (150 mg) or intravenously four times per year (3 mg) for women with postmenopausal osteoporosis and increased risk of fractures. Although a risk reduction of vertebral fractures has been shown, no data on risk reduction of hip fractures exist (Chesnut et al., 2004[27]).

Hypersensitivity, hypocalcemia, and severe renal impairment (glomerular filtration rate ≤ 35ml for alendronate and zoledronic acid, and ≤ 30 ml for ibandronate and risedronate), pregnancy, lactation, and pathologies of the esophagus are contraindications for the use of bisphosphonates. The treatment of vitamin D deficiency and hypocalcemia is mandatory before the start of bisphosphonates.

Osteonecrosis of the jaw and atypical fractures of the femoral bone are rare side effects of bisphosphonates (Mucke et al., 2016[81]).

#### Denosumab

RANKL was established as target for the regulation of osteoclast generation based on studies in knock-out mice (Theill et al., 2002[121]). Denosumab, a monoclonal antibody against RANKL, is given subcutaneously 60 mg twice a year. It is approved for postmenopausal women and men at advanced risk for fractures and has been shown to reduce the risk for vertebral and hip fractures. It is not recommended in subjects under the age of 18 years; skin infections and hypocalcemia are known side effects. For the rare side effect of jaw necrosis, the same precautions are taken as with bisphosphonate treatment (Cummings et al., 2009[35]). Because of an increased rate of vertebral fractures after discontinuing denosumab, a transition to an alternative treatment should be initiated after ending of denosumab therapy (Cummings et al., 2018[34]). 

#### SERMs 

Selective estrogen receptor modulators (SERMs) are synthetic estrogen receptor ligands that are approved for both the prevention and treatment of osteoporosis in postmenopausal women. They induce a different response than estradiol and enhance osteoclast apoptosis. SERMs approved for the treatment of osteoporosis are raloxifene, lasofoxifene, and bazedoxifene. In postmenopausal women raloxifene reduced the risk of vertebral fractures by 30 % in patients with a prior vertebral fracture and by approximately 55 % in patients without a prior vertebral fracture over three years; nevertheless, raloxifene did not protect against nonvertebral or hip fractures (Khosla, 2010[67]). Cummings and coworkers in 2010[35], found that in postmenopausal women with osteoporosis, lasofoxifene at a dose of 0.5 mg per day was associated with reduced risks of nonvertebral and vertebral fractures, but an increased risk of venous thromboembolic events. In a systematic review, a significant risk reduction of nonvertebral fractures and vertebral fractures in postmenopausal women was demonstrated for all three substances (Barrionuevo et al., 2019[9]). Due to its ability to prevent breast cancer, SERMs are considered as a treatment option for the prevention and treatment of osteoporosis in women with high risk of osteoporotic fractures but without a previous history of thromboembolic disease (D'Amelio and Isaia, 2013[38]).

#### Hormone replacement therapy

Estrogens inhibit bone resorption directly by stimulating the apoptosis of osteoclasts and suppressing the apoptosis of osteoblasts and osteocytes (Bagger et al., 2004[8]). Primarily they were prescribed for the relief of postmenopausal symptoms like insomnia, sweating, mood disturbances, and vaginal dryness. In the Women's Health Initiative studies in elderly women hormone replacement therapy increased the risk of breast cancer, cerebrovascular, and thromboembolic diseases. In post hoc analyses, these side effects were not found in the group of women that used estrogen only. Currently hormone replacement therapy is approved by the U.S. Food and Drug Administration for the prevention of osteoporosis and treatment of menopausal symptoms - definitely not as a first line therapy for osteoporosis (Chen et al., 2019[25]). In the light of the unfavorable risk/benefit balance the guidelines of Dachverband Osteologie (DVO; http://dv-osteologie.org/osteoporose-leitlinien) recommend the use of estrogens/gestagens only in case of intolerance with other osteoporosis drugs.

#### Osteoanabolic therapy

In contrast to the action of antiresorptive agents (like bisphosphonates, denosumab, estrogens, and raloxifene), osteoanabolic drugs activate bone formation due its effects on osteoblasts (Tabacco and Bilezikian, 2019[116]). Osteoanabolic therapy is underutilized for cost concerns and underestimation of the clinical benefit, since hip fractures in intervention trials were not assessed as a primary endpoint. In fact, teriparatide and romosozumab are more effective in reducing the risk of vertebral and nonvertebral fractures than antiresorptive drugs (Cosman, 2020[32]). Safety concerns relate to an increased risk of osteosarcoma in rodent trials on teriparatide; this effect was not seen in trials with primates. Nevertheless, these substances are not recommended in patients with prior radiation involving the skeleton or a positive family history of osteosarcoma (Gilsenan et al., 2018[55]).

**Abaloparatide** and **teriparatide** are approved for the treatment of osteoporosis in the United States. In Europe, because of cardiovascular side effects of abaloparatide, only teriparatide is available. They are recombinant analogues of parathyroid hormone (PTH), a key regulator of calcium homeostasis, and increase bone formation and bone mass. Teriparatide is approved for osteoporotic women and men at high risk for fracture and for osteoporosis associated with long-term glucocorticoid therapy. **Intermittent** injection of a low dose promotes bone anabolism more than the release of calcium and phosphorus by osteoclasts (as in the case of **continuous** action of high levels of parathyroid hormone). Treatment with 20 µg teriparatide daily subcutaneously provided a risk reduction of vertebral fractures by 65 % and nonvertebral fractures by 53 % in osteoporotic patients after a period of 18 month (Chen et al., 2015[24]; Neer et al., 2001[84]). Teriparatide is used for a maximum of two years following another specific anti-osteoporotic treatment, since safety and efficacy have been established only for this period. 

**Romosozumab** is an antibody against sclerostin, a secreted glycoprotein that acts as key negative regulator of bone formation. Sclerostin is encoded by the SOST gene and in bone is specifically expressed by osteocytes. Binding of sclerostin to LPR 5 and 6 prevents activation of canonical Wnt signaling in bone, resulting in decreased bone formation (for review see Kerschan-Schindl, 2020[66]). Romosozumab given in monthly doses of 210 mg subcutaneously in postmenopausal women with osteoporosis and a fragility fracture reduced the risk of further vertebral fractures by 48 % versus alendronate alone and the risk of hip fractures by 38 % (Saag et al., 2017[107]). This anti-sclerostin antibody stimulates bone formation and at the same time inhibits bone resorption. In 2016 Cosman et al. reported on 7180 women with a low bone mineral density (t-score between -2,5 to -3,5) that were treated with 210 mg once monthly subcutaneously versus placebo following a therapy with denosumab after one year of treatment. In comparison with placebo, the risk of further vertebral fractures was 73 % lower. The rate of nonvertebral fractures was lower as well (1,6 % versus 2,1 % in the placebo arm of the study) (Cosman et al., 2016[33]). Because this remedy just recently has been approved in Europe, further considerations are mentioned under “future prospects“.

#### Combination therapy

Osteoporosis therapy (apart from calcium and vitamin D supplementation) is administered as monotherapy. Nevertheless, some clinical trials evaluated combination therapies of osteoanabolic and antiresorptive agents to increase the effect on bone formation and fracture risk reduction. In these studies beneficial effects on bone mineral density were seen, but due to deficiencies in the study design (lack of a monotherapy arm, missing evaluation of fracture risk, comparable outcomes with monotherapy) combination therapies neither are recommended in general nor approved by the health care systems (Anastasilakis et al., 2020[4]).

#### Long term treatment and monitoring

Discontinuation of teritaratide is followed by a loss of bone mineral density, but further gain can be achieved with a sequential antiresorptive therapy. Antiresorptive agents have rare side-effects like osteonecrosis of the jaw and atypical fractures of the femur in a time dependent manner. Therefore, discontinuation and reevaluation is recommended after 3-5 years (Whitaker et al., 2012[131]); it is advisable to adapt the treatment regimes in the long-term therapy of osteoporosis. A weakness of all trials on combination therapies is the use of a surrogate marker - bone mineral density - instead of fracture risk to assess treatment efficacy. Comparing all possible options of sequences, teriparatide followed by denosumab was most effective for bone mineral density gain. The most often used sequence of antiresorptive agent or osteoanabolic agent following an antiresorptive treatment was moderately effective in that respect. Denosumab therapy following an antiresorptive or teriparatide treatment is even more effective than the sequence with another antiresorptive agent (Miller et al., 2020[79]). Based on studies of various sequences of osteoanabolic and antiresorptive drugs, the start with an osteoanabolic drug followed by antiresorptive drugs is most effective to achieve greatest hip bone mineral density gains (Cosman, 2020[32]).

#### Nonpharmacologic treatment

Following a systematic review of Coronado-Zarco et al. (2019[31]), nonpharmacologic treatment can be summarized in 14 points for clinical practice (see Table 2(Tab. 2); References in Table 2: Al-Bashaireh and Alqudah, 2020[2]; Avenell et al., 2016[7]; Bischof-Ferrari et al., 2004[13]; Bougioukli et al., 2019[18]; Chau et al., 2020[23]; Compston et al., 2017[30]; Dobnig, 2011[44]; Ganz and Latham, 2020[52]; Gillespie et al., 2012[54]; Holick, 2007[60]; Tinetti, 2003[122]; Vandenbroucke et al,. 2017[126]).

#### Adherence

Adherence means the extent to which patients follow instructions or medical prescriptions (Osterberg and Blaschke, 2005[89]). Patients take medication on their own decision based on estimated risk balanced with their perceived benefit. This decision is individually interpreted based on the quintessence of information they receive. Many patients suffering from osteoporosis do not feel any symptoms until the first fracture occurs and prefer not to take medication without symptoms. Even in patients after a hip fracture a low adherence to oral bisphosphonates has been reported. Nonadherence was found in a group of 368 patients with a mean age of 85 years in 65 % of cases. Multivariate analysis found a poorer functional status prior to treatment as risk factor (Gamboa et al., 2018[50]). Biases of decisions depend on their emotions (too optimistic or too negative in case of depressed mood), trust in the health care system, gender, and on sociocultural factors. To improve adherence to prescribed drugs against osteoporosis, a clear understanding of patient's attitude and thoughts is required to intervene with tailored information and motivational interventions (Silverman and Gold, 2018[112]).

#### Polypharmacy

Polypharmacy - the use of more than 5 drugs or the use of potentially inappropriate medication - is associated with an increased risk of hip fracture. This is the result of a case-control study on 1003 female patients (on average 71 years of age) with osteoporosis and a hip fracture after adjustment for confounders. The odds ratio for patients using 5-10 drugs was 1,84 (confidence interval 1,49-2, 28) and 2,50 (1,36-4,62) for patients using more than 10 drugs. This holds true after adjustment for the use of glucocorticoids and benzodiazepines (Park et al., 2019[93]). A comedication with proton pump inhibitors is associated with lower trabecular bone density in older individuals. Many hypotheses on the mechanism how proton pump inhibitors harm bone exist: the interference with bisphosphonates, decreased bioavailability of micronutrients and vitamins (calcium, magnesium, vitamin B-12) necessary for bone metabolism due to hypochlorhydria, hypergastrinemia causing reduced calcium bioavailability and an increase of PTH levels (Maggio et al., 2013[78]). Fall risk inducing drugs are of special concern in patients with a history of high risk of falls. A reduction of the number of these drugs, a reduction of their dose, or replacement has been shown to decrease the risk of osteoporotic fractures (Chen et al., 2014[26]). 

#### Future prospects

Aside from sclerostin also other components of the Wnt signaling pathway are important targets of new drugs for osteoporosis. **Dickkopf-1** is an inhibitor of the aforementioned signaling pathway and is deregulated in glucocorticoid induced bone loss and in arthritis. It could be a future target in types of osteoporosis that are predominantly associated with inhibited bone formation (Chen et al., 2019[25]).

The protease** Cathepsin K** which is produced by osteoclasts is another target for the development of an osteoporosis-specific medication. Odanacatib, an inhibitor of cathepsin K reached phase 2 and 3 trials in postmenopausal women (Boonen et al., 2012[17]). Nevertheless, the development of odanacatib was stopped due to cardiovascular side effects. Hopefully, another way of cathepsin K inhibition will be developed in the future.

Very promising is the development of senolytic drugs. Aged subjects very often suffer from several chronic diseases that may require the prescription of a relatively high number of medications, potentially leading to the dilemma of polypharmacy (see above). In this regard, drugs that target cellular senescence (senolytics, senostatics) represent an interesting novel therapeutic approach, since they are expected to interfere with multiple age associated diseases (Kang, 2019[63]; Khosla et al., 2018[68]). As mentioned before, in a preclinical setting, the **senolytic** drug ruxolitinib was effective in the prevention of age-related deterioration of bone microstructure (Farr et al., 2017[47]).

## Acknowledgements

This work was supported in part by a LÓreal Österreich scholarship awarded to UAS (# 186/14-tww).

## Conflict of interest

Katharina Kerschan-Schindl has received research support and/or remuneration from Amgen GmbH, Lilly GmbH, Merck, Sharp and Dohme GmbH, Roche Austria, and Servier Austria. Peter Pietschmann has received research support and/or honoraria from Amgen GmbH, Biomedica GmbH, DePuySynthes, Eli Lilly GmbH, Fresenius Kabi Austria, Meda Pharma/Mylan GmbH, Shire Austria GmbH, TAmiRNA GmbH and UCB Pharma. Peter Dovjak has received honoraria from Daiichi Sankyo Austria GmbH, Ratiopharm GmbH, Novartis Austria and Sanofi Austria. All other authors have no conflict of interest to declare.

## Figures and Tables

**Table 1:**
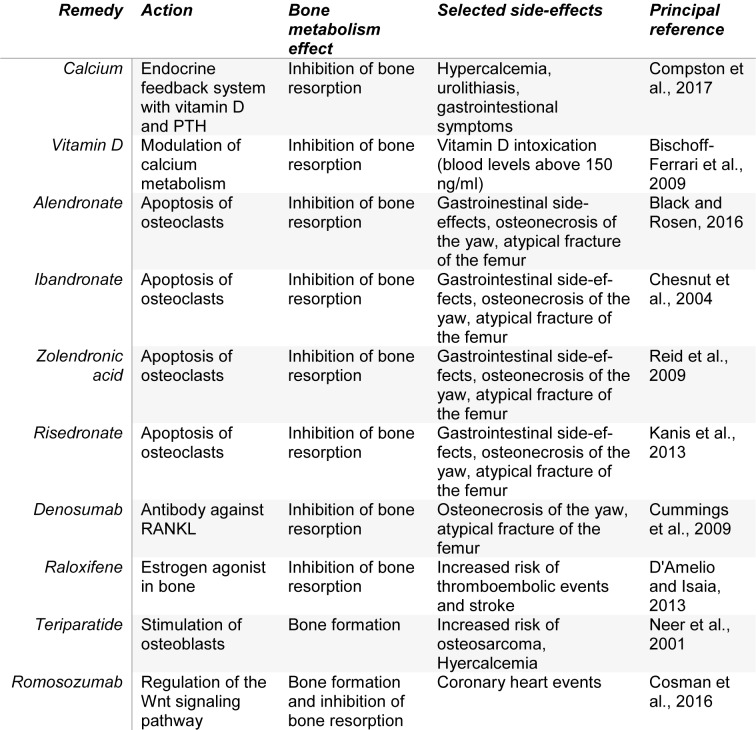
Selected treatment options for osteoporosis mentioned in this review

**Table 2:**
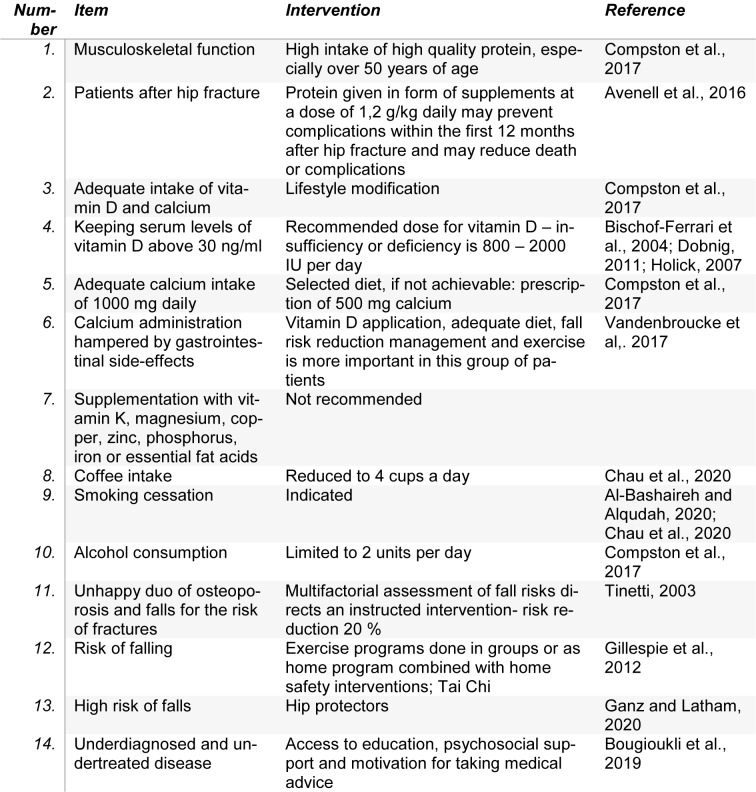
Nonpharmacologic treatment
